# A standardized citation metrics author database annotated for scientific field

**DOI:** 10.1371/journal.pbio.3000384

**Published:** 2019-08-12

**Authors:** John P. A. Ioannidis, Jeroen Baas, Richard Klavans, Kevin W. Boyack

**Affiliations:** 1 Departments of Medicine, Health Research and Policy, Biomedical Data Science, and Statistics and Meta-Research Innovation Center at Stanford (METRICS), Stanford University, Stanford, California, United States of America; 2 Research Intelligence, Elsevier B.V., Amsterdam, the Netherlands; 3 SciTech Strategies, Inc., Wayne, Pennsylvania, United States of America; 4 SciTech Strategies, Inc., Albuquerque, New Mexico, United States of America

## Abstract

Citation metrics are widely used and misused. We have created a publicly available database of 100,000 top scientists that provides standardized information on citations, h-index, coauthorship-adjusted hm-index, citations to papers in different authorship positions, and a composite indicator. Separate data are shown for career-long and single-year impact. Metrics with and without self-citations and ratio of citations to citing papers are given. Scientists are classified into 22 scientific fields and 176 subfields. Field- and subfield-specific percentiles are also provided for all scientists who have published at least five papers. Career-long data are updated to end of 2017 and to end of 2018 for comparison.

## Abstract 2

Citation metrics are widely used and misused. This Community Page article presents a publicly available database that provides standardized information on multiple citation indicators and a composite thereof, annotating each author according to his/her main scientific field(s).

Use of citation metrics has become widespread but is fraught with difficulties. Some challenges relate to what citations and related metrics fundamentally mean and how they can be interpreted or misinterpreted as a measure of impact or excellence [1]. Many other problems are of a technical nature and reflect lack of standardization and accuracy on various fronts. Several different citation databases exist, many metrics are available, users mine them in different ways, self-reported data in curriculum vitae documents are often inaccurate and not professionally calculated, handling of self-citations is erratic, and comparisons between scientific fields with different citation densities are tenuous. To our knowledge, there is no large-scale database that systematically ranks all the most-cited scientists in each and every scientific field to a sufficient ranking depth; e.g., Google Scholar allows scientists to create their profiles and share them in public, but not all researchers have created a profile. Clarivate Analytics provides every year a list of the most-cited scientists of the last decade, but the scheme uses a coarse classification of science in only 21 fields, and even the latest, expanded listing includes only about 6,000 scientists (https://hcr.clarivate.com/worlds-influential-scientific-minds), i.e., less than 0.1% of the total number of people coauthoring scholarly papers. Moreover, self-citations are not excluded in these existing rankings.

We have tried to offer a solution to overcome many of the technical problems and provide a comprehensive database of a sufficiently large number of most-cited scientists across science. Here, we used Scopus data to compile a database of the 100,000 most-cited authors across all scientific fields based on their ranking of a composite indicator that considers six citation metrics (total citations; Hirsch h-index; coauthorship-adjusted Schreiber hm-index; number of citations to papers as single author; number of citations to papers as single or first author; and number of citations to papers as single, first, or last author) [2].

The methodology behind the composite indicator has been already extensively described along with its strengths and residual caveats in [2]. We offer two versions of the database. One version (supplementary Table S1, http://dx.doi.org/10.17632/btchxktzyw.1#file-ad4249ac-f76f-4653-9e42-2dfebe5d9b01) is calculated using Scopus citation data over 22 years (from January 1, 1996 until December 31, 2017; complete data for 2018 will not be available until later in 2019). For papers published from 1960 until 1995, the citations received in 1996–2017 are also included in the calculations, but the citations received up to 1995 are not. Therefore, this version provides a measure of long-term performance, and for most living, active scientists, this also reflects their career-long impact or is a very good approximation thereof. In order to assess the robustness and validity of the calculations, they have been replicated on a second, independent platform and a data set with a slightly different timestamp (less than one month difference). Correlations between the two independent calculations for the composite indicator (r = 0.983) and number of papers (r = 0.991) for the top 1,000,000 authors confirm the calculations are accurate and stable.

The other version (supplementary Table S2, http://dx.doi.org/10.17632/btchxktzyw.1#file-b9b8c85e-6914-4b1d-815e-55daefb64f5e) is calculated using data for citations in a single calendar year, 2017. It provides a measure of performance in that single recent year. Therefore, it removes the bias that may exist in comparing scientists with long accrual of citations over many years of active work versus younger ones with shorter time frame during which they may accumulate citations because it focuses on citation accrual only during a single year.

The constructed database shows, for each scientist, the values for each of the six metrics that are used in the calculation of the composite as well as the composite indicator itself, and all indicators are given with and without self-citations. Institutional affiliation and the respective country are inferred based on most recent publications according to the Scopus data as of May 2018. Therefore, only one affiliation is provided even though scientists may have worked in several institutions. Nevertheless, all their work in different institutions is all captured within their author record.

Extreme self-citations and “citation farms” (relatively small clusters of authors massively citing each other’s papers) make citation metrics spurious and meaningless, and we offer ways to identify such cases. We provide data that exclude self-citations to a paper by any author of that paper and, separately, data including all citations, e.g., if a paper has 12 authors and it has received 102 citations, but 24/102 have as a (co)author at least one of these 12 authors of the original paper, only 102 − 24 = 78 citations are counted. Among the top 100,000 authors for 1996–2017 data, the median percentage of self-citations is 12.7%, but it varies a lot across scientists (interquartile range, 8.6%–17.7%, full range 0.0%–93.8%). Among the top 100,000 authors for the 2017 single-year data, the median percentage of self-citations is 9.2% (interquartile range, 4.8%–14.7%, full range 0.0%–98.6%). With very high proportions of self-citations, we would advise against using any citation metrics since extreme rates of self-citation may herald also other spurious features. These need to be examined on a case-by-case basis for each author, and simply removing the self-citations may not suffice [3]. Indicatively, among the top 100,000 authors for 1996–2017 and 2017-only data, there are 1,085 and 1,565 authors, respectively, who have >40% self-citations, while 8,599 and 8,534 authors, respectively, have >25% self-citations.

We also provide data on the number of citing papers and on the ratio of citations divided by the number of citing papers. 5,709 authors in the career-long data set and 7,090 in the single-year data set have a ratio over 2. High ratios deserve more in-depth assessment of these authors. Sometimes, this may reflect that it is common for a small number of papers of the same author to be cited together. Alternatively, they may point to situations of spurious “citation farms.”

For each scientist, we provide the most common scientific field and the two most common scientific subfields of his/her publications, along with the percentage for each. All science is divided into 22 large fields (e.g., Clinical Medicine, Biology), and these are further divided into 176 subfields according to the Science-Metrix journal classification system [4] (http://science-metrix.com/?q=en/classification). Thus, users can rank scientists according to each of the six metrics or the composite indicator and can limit the ranking to scientists with similar scientific field or top subfield for different levels of desired similarity.

A separate file (supplementary Table S3, http://dx.doi.org/10.17632/btchxktzyw.1#file-e30a1e62-daf4-49f1-b1ca-484a979f6500) lists the total number of authors in Scopus who have published at least five papers and breaks this down by their most common area of publications (for the 22 fields and 176 subfields mentioned above). A total of 6,880,389 scientists have published at least five papers. Because each of the top 100,000 authors can be assigned to the most common field or subfield to which his/her work belongs, a ranking can be obtained among authors assigned to the same main area based on what journals they publish in; e.g., suppose a scientist is ranked 256 in some particular metric among the 120,051 scientists in the subfield of immunology. Therefore, the scientist is in the top 0.21% (256/120,051) of authors by that metric in immunology.

For all 6,880,389 scientists, Table 1 shows the career-long 25th, 50th, 75th, and 90th percentile of total citations and composite citation index according to each of the 22 fields. Table S3 provides the same information (along with 95th and 99th percentiles) for each of the 176 subfields as well. Thus, one can see the relative citation density of different fields. Moreover, any scientist who has published at least five papers can be ranked against these standard percentiles in his/her field or subfield based on his/her citation data from Scopus.

**Table 1:** Percentiles of total citations and composite citation metric for each of 22 large scientific fields, career-long data (citations from 1996–2017). Total citations include self-citations.

Scientific field	Authors	Percentile, total citations				Percentile, composite index			
		25th	50th	75th	90th	25th	50th	75th	90th
Agriculture, Fisheries, & Forestry	232,801	32	90	255	671	0.997	1.418	1.892	2.394
Built Environment & Design	36,534	17	51	143	370	0.953	1.344	1.821	2.335
Enabling & Strategic Technologies	475,142	23	75	233	678	0.890	1.330	1.807	2.300
Engineering	436,723	18	56	174	499	0.896	1.316	1.794	2.314
Information & Communication Technologies	339,284	20	60	193	574	0.970	1.380	1.862	2.383
Communication & Textual Studies	20,292	12	32	91	240	1.141	1.542	1.995	2.430
Historical Studies	25,277	16	40	105	263	1.138	1.568	2.012	2.429
Philosophy & Theology	13,861	12	32	87	217	1.145	1.558	2.003	2.453
Visual & Performing Arts	3,717	7	17	40	83	0.985	1.316	1.680	1.998
Economics & Business	108,277	28	83	258	708	1.191	1.651	2.194	2.730
Social Sciences	119,260	20	56	158	423	1.159	1.606	2.114	2.615
General Science & Technology	69,789	14	41	122	399	0.735	1.030	1.392	1.760
General Arts, Humanities, & Social Sciences	4,091	11	28	70	158	1.026	1.403	1.810	2.192
Biomedical Research	626,753	68	212	641	1,769	1.095	1.598	2.111	2.660
Clinical Medicine	2,113,734	41	141	467	1,430	0.935	1.420	1.979	2.568
Psychology & Cognitive Sciences	96,159	41	128	403	1,198	1.189	1.641	2.198	2.842
Public Health & Health Services	141,162	31	92	273	785	0.988	1.427	1.949	2.520
Biology	236,108	47	140	426	1,178	1.151	1.603	2.125	2.686
Chemistry	506,526	45	129	362	989	1.057	1.503	1.967	2.467
Earth & Environmental Sciences	223,246	40	126	405	1,192	1.096	1.562	2.120	2.709
Mathematics & Statistics	96,619	18	52	162	457	1.049	1.503	2.059	2.596
Physics & Astronomy	667,255	38	128	480	1,741	1.022	1.495	2.042	2.615
Unassigned*	287,779	2	7	18	42	0.463	0.672	0.985	1.302
TOTAL	6,880,389	29	102	346	1,077	0.946	1.420	1.951	2.513

In order to calculate the c (composite) indicator, any scientist may use the formula $\text{c}=\frac{\text{ln}\left(nc9617+1\right)}{\text{ln}\left(nc9617max+1\right)}+\frac{\text{ln}\left(h17+1\right)}{\text{ln}\left(h17max+1\right)}+\frac{\text{ln}\left(hm17+1\right)}{\text{ln}\left(hm17max+1\right)}+\frac{\text{ln}\left(ncs+1\right)}{\text{ln}\left(ncsmax+1\right)}+\frac{\text{ln}\left(ncsf+1\right)}{\text{ln}\left(ncsfmax+1\right)}+\frac{\text{ln}\left(ncsfl+1\right)}{\text{ln}\left(ncsflmax+1\right)}$, where nc9617 is the total number of citations, h17 is the h-index, hm17 is the Schreiber coauthorship-adjusted hm index, ncs is the number of citations to papers as a single author, ncsf is the number of citations to papers as single or first author, and ncsfl is the number of citations to papers as single, first, or last author. The maximum values for these components of the composite indicator are nc9617max = 259,310, h17max = 222, hm17max = 103.9811, ncsmax = 135,334, ncsfmax = 149,125, and ncsflmax = 163,476. For the same percentiles on career-long total citation and composite indicator data split according to 176 subfields, see Table S3.

*Unassigned scientists have no published items that can be assigned to any field. Typically, they have published very few items, and these may be in conference proceedings or journals that are not included in the Science-Metrix classification system.

The data in the Table include all authors who have published at least five items that are classified by Scopus as “Articles,” “Reviews,” or “Conference Papers.”

Existing ranking systems typically focus on single fields (e.g., ranking of authors in economics is performed by https://ideas.repec.org/top/) and use numbers of papers and total citations rather than multiple metrics. They also do not account for self-citation phenomena. Nevertheless, our databases still have limitations that have been discussed in detail previously in describing the methodology behind the composite indicator [2]. We should also caution again that citations from before 1996 are missing from our analysis. Overall, whole-career metrics place young scientists at a disadvantage. Single-year metrics remove much of this problem, although again, younger scientists have fewer years of publication history and thus probably fewer papers that can be cited in 2017. We have included the year of first (earliest) publication and the year of last (more recent) indexed publication of each author.

Publications of the scientists are extracted from the Scopus database using the author profiles, which are formed by a combination of curated profiles and profiles generated by an “author profiling” algorithm [5]. The reported precision and recall by Scopus in 2017 was 98% precision (i.e., on average, 98% of publications merged in a profile belong to one and the same person) at an average recall of 93.5% (i.e., on average, 93.5% of all publications of the same person are merged into one profile); the evaluation used a manual assessment of a sample of >6,000 authors for which the full publication history was collected and compared to what is available in the Scopus profiles. The precision/recall is higher as of April, 2019 at 99.9% and >94%, and the gold set used is also larger now, with >10,000 author records. Nevertheless, a few scientists still have their work split into multiple author records in Scopus; however, even then, one record usually carries the lion’s share of citations. We examined in depth a random sample of 500 author records among the top 1,000,000 records according to the 1996–2017 composite indicator, and we found 13 authors who had been split into two records each. It is possible that the most-cited/most-productive authors may have a higher chance of having split records. Among the top 150 in terms of composite indicator for 1996–2017, we found 20 who had two records and three who had three records among the top 1,000,000 records. However, in all cases, the top record captured the large majority of the citations, and for 11/23, the extra record(s) were not even among the top 100,000. Some other scientists with the same name may have been merged in the same record, but overall, disambiguation in Scopus has improved markedly in this regard, and major errors of this sort are currently very uncommon. They may be more common still for some Chinese and Korean names. Inappropriate merging may also be suspected when the top subfields are not contiguous, e.g., diabetes and particle physics.

Some citation indicators such as the h-index are highly popular, but all single indicators have shortcomings. For practical purposes, it is usually desirable to have a set of bibliometric indicators, each emphasizing a different aspect of the scientific impact of a scientist [6]. We offer the means to practice routinely such an approach. Of note, the six components of the composite indicator are not orthogonal but have correlations among themselves. Some bibliometrics experts may not favor composites that include correlated metrics and may prefer to inspect each one of them independently. Our databases also allow this approach.

The data sets that we provide also allow placing scientists in reference standards of almost two hundred fields. Still, some scientists may work in very small sub-subfields that may have different citation densities. Moreover, for very early career scientists, any citation metrics would have limited use since these researchers may not have published much yet and their papers would not have time to accrue citations.

A citation database is most useful when it can be regularly updated. We also provide here data that have been updated with an annual interval. We repeated the same exact analyses for career-long data until the end of 2018 (as opposed to the end of 2017) using a timestamped Scopus data set released on April 22, 2019. The data on the top-100,000–ranked scientists are provided in supplementary Table S4 (http://dx.doi.org/10.17632/btchxktzyw.1#file-bade950e-3343-43e7-896b-fb2069ba3481). As one can see, the correlation between the two data sets is extremely high, and the vast majority of scientists do not change their ranking much. As an illustrative example, supplementary Table S5 (http://dx.doi.org/10.17632/btchxktzyw.1#file-5d904ef8-fc87-4dbf-aaa7-ad33db9ac561) provides the ranking for a random sample of 100 authors sampled from those who were in the top 100,000 based on the composite index excluding self-citations. 93 of the 100 were among the top 100,000 in both assessments. Another five were very close to the top 100,000 with one assessment and at the lower end of the top 100,000 in the other assessment. Another two with modestly larger differences still did not shift by much in terms of their percentile ranking across all authors, with changes of 1% and 2% on the percentile ranking, respectively. Both of these changes were due to corrections in which papers are included in the author record rather than simply accrual of citations. For the vast majority of scientists, it is likely that percentile ranking may take many years to change substantially; therefore, the current databases that we have compiled can be used meaningfully for several years by the wider community before a new update is needed. We provide the databases as spreadsheets in Mendeley Data for entirely open, free public use. Instead of creating a formulaic website, spreadsheets can be downloaded, searched, and tailored for analyses by scientists in whatever fashion they prefer. Moreover, the percentile information could be used for placing a field-specific ranking for any scientist, not just the top 100,000.

We hope that the availability of standardized, field-annotated data will help achieve a more nuanced use of metrics, avoiding some of the egregious errors of raw bean-counting that are prevalent in misuse of citation metrics. Citation metrics should be used in a more systematic, less error-prone and more relevant, context-specific, and field-adjusted way and also allowing for removal of self-citations and detection of citation farms.

Citation analyses for individuals are used for various single-person or comparative assessments in the complex reward and incentive system of science [7]. Misuse of citation metrics in hiring, promotion or tenure decision, or other situations involving rewards (e.g., funding or awards) takes many forms, including but not limited to the use of metrics that are not very informative for scientists and their work (e.g., journal impact factors); focus on single citation metrics (e.g., h-index); and use of calculations that are not standardized, use different frames, and do not account for field. The availability of the data sets that we provide should help mitigate many of these problems. The database can also be used to perform evaluations of groups of individuals, e.g., at the level of scientific fields, institutions, countries, or memberships in diversely defined groups that may be of interest to users. Linkage to other author-based databases in the future may enhance the potential for further use in meta-research evaluations [8]. We discourage raw comparisons of scientists across very different fields. We cannot emphasize enough that use of these metrics needs to be prudent. Authors who detect errors in the entered data should contact Scopus to correct the respective entries and author records. We also welcome suggestions for more generic improvements that may augment the utility of the shared resource that we have generated.
